# The implementation of an innovative just‐in‐time educational huddle for faculty development of academic hospitalists

**DOI:** 10.1002/jhm.70436

**Published:** 2026-09-03

**Authors:** Donna Coetzee, Suman Pal, Elizabeth Davis, Andrew P. J. Olson, Melissa Plesac

**Affiliations:** ^1^ Department of Medicine Division of Hospital Medicine, University of Minnesota Medical School Minneapolis Minnesota USA; ^2^ Department of Medicine Division of Hospital Medicine, Duke University School of Medicine Durham North Carolina USA

## Abstract

Academic hospitalists supervising residents on inpatient wards face the dual challenge of managing changing patient complexity and learner needs. Resident experience is highly dependent on supervising faculty; however, traditional faculty development often fails to respond to real‐time challenges of clinical teaching. To provide just‐in‐time faculty development, we implemented a weekly educational huddle for hospitalists rotating on resident ward teams using a Community of Practice model. The huddle emphasized peer coaching and discussion of real‐time challenges. In this perspective article, we share that our findings showed high participation and self‐reported improvements in teaching, feedback, coaching, efficiency, and sense of belonging. This innovation represents a feasible approach to faculty development in hospital medicine.

## INTRODUCTION

Inpatient rotations are a core requirement of Internal Medicine residency training.^1^ Medicine wards rotations represent a major component of this experience. However, residents’ experience on these rotations can vary widely in terms of workload, patient complexity, and dedicated teaching. Moreover, residents’ learning experience is reliant on their supervising attending's engagement with teaching.^2^ However, attendings’ teaching approaches can vary in terms of level of supervision, expectations, level of entrustment, and teaching expertise.^3^ Therefore, ensuring a consistent, high‐quality educational experience can be challenging.

Faculty development for attendings is important to address these challenges. Traditional faculty development programs, including workshops, lectures, conferences, and courses are resource‐intensive and occur outside the clinical environment. Therefore, they may be poorly positioned to address real‐time challenges that arise during inpatient service, where team dynamics, patient complexity, and workflow demands frequently evolve. Approaches that support peer learning within the clinical environment may be better positioned to address these challenges, and a community of practice (CoP) model of faculty development can present a viable alternative. CoP is a persistent, sustained social network of individuals who share and develop an overlapping knowledge base, set of beliefs, values, history, and experiences focused on a common practice and/or mutual enterprise.^4^ A CoP model offers several benefits over traditional models for faculty development, including responsiveness to immediate needs, allowing for iterative learning, and improving belonging through meaningful participation.^5^, ^6^, ^7^

We implemented an innovative weekly educational huddle for developing skills for hospitalists supervising residents on medicine ward rotations. In this article, we discuss the design and implementation of our educational huddle and report early outcomes and implications for academic hospital medicine faculty using a CoP lens.

### Strategy/Innovation

At our urban academic medical center, we have five medicine ward teams with residents. Each team is supervised by one attending hospitalist and consists of one senior resident, either working independently or with one intern. Additional undergraduate medical education (UME) learners also often join the team. Division of Hospital Medicine (DHM) faculty rotate on teaching services in 1‐week blocks. Faculty spend up to 25%–50% of their clinical time on learner teams with the rest being on direct service lines or with advanced practice providers (APPs), though it varies based on how much clinical time each faculty has and whether they do specialty services (e.g., procedures).

The aim of the educational huddle was to provide a structured opportunity for peer‐to‐peer coaching among hospitalists focused on clinical teaching. Importantly, the huddle was not to focus on specific resident or medical student challenges, but to provide more global, faculty‐driven, just‐in‐time teaching support. Each huddle consisted of a 15‐to‐30‐min on‐site meeting held on the afternoon of the second day of service. This timing gave hospitalists 2 days of accumulated teaching experiences, questions, and challenges to bring to the discussion, while leaving sufficient time in the remaining service week to implement suggested changes. Participants invited included the five hospitalists on service, a representative each from residency leadership and DHM educational leadership, and a chief resident. While attendance was not mandatory, there was a long‐term plan to tie consistent failure to attend to a loss of teaching privileges.

To facilitate discussion and maintain efficiency, we developed a script for each hospitalist to review at the start of the meeting (Supporting Information S1: Appendix 1). This script was aimed at eliciting challenges that commonly occur in the first few days of service. After this script, the remaining time was intentionally left unstructured to allow for open discussion about issues raised.

To monitor implementation and assess perceived value of the huddles, we designed two tools. The first was a brief exit instrument (Supporting Information S1: Appendix 2) that was filled out by a chief resident or leadership representative at the end of each huddle. This tracked meeting duration, attendance, and topics discussed. Four months after implementation, we began administration of a cross‐sectional survey (Supporting Information S1: Appendix 3) to collect data from hospitalists on engagement, relevance, and impact of the huddle. This survey was sent via email and provided as a link at the end of huddle sessions. Participation was voluntary and not anonymous. The survey was live for 3 months of data collection. Google Forms was used for both the exit instrument and survey. We performed descriptive analysis in Excel on survey results to evaluate the feasibility, acceptability, and early outcomes of the educational huddles. This project was determined to not be human subjects research by the University of Minnesota Institutional Review Board.

## RESULTS

At the time of writing, 37 sessions had been conducted. After an initial pilot period, we began using the exit instrument and had recorded data from 20 sessions at the time of analysis. The median duration was 23 min (15–40 min). Of the five hospitalists on service each week, a mode of four were present at each huddle. During this period, 65 hospitalists within DHM completed at least 1 week on a teaching service. Twenty‐four faculty responded, yielding a survey response rate of 37%.

Analysis of exit instrument data demonstrated that efficiency and team dynamics were the most frequently discussed topics, addressed in 90% and 85% of huddles, respectively (Table 1). Challenges related to supporting struggling learners were also common, including issues related to medical knowledge or clinical care (35%) and professionalism (5%). Additional topics included feedback, case‐specific issues, and teaching strategies. Nearly half of huddles included discussion of other timely or emerging issues (e.g., supporting new attendings, use of technology like workstations‐on‐wheels or artificial intelligence, discharge preparation).

**Table 1:** Frequency distribution of exit instrument and faculty survey responses.

Domain	Exit instrument item	Result (*N* = 20)	Response reported
Topics of discussion	Efficiency	90.00% (18)	Percentage (number) of educational huddles where topic was discussed
	Team Dynamics	85.00% (17)	
	Feedback	40.00% (8)	
	Struggling Learner—Medical Knowledge or Clinical Care	35.00% (7)	
	Struggling Learner—Professionalism	5.00% (1)	
	Patient or Clinical Issue	30.00% (6)	
	Teaching on Wards	25.00% (5)	
	Other: New attending, WOW use, AI use, off service learners, working with medical students, responding to learner emotions, discharge prep, distribution of patients between learners	45.00% (9)	

Domain	Faculty survey question	Result (*N* = 24)	Response reported
Engagement in Huddles	When you attended, how often did you seek advice from the group?	58.33% (14)	Responses of “more than half the time” or “always”
	When you attended, how often did you give advice on a topic raised in the group?	58.33% (14)	
	When you attended, how often did someone seek advice on an issue that you have also encountered?	79.17% (19)	
Faculty views of a structured educational huddle	The educational huddles are a valuable use of my time during teaching team service.	95.83% (23)	Agreement score of 4 or 5 on a 5‐point Likert scale
	Participating in educational huddles has helped me understand the expectations of attendings on teaching service.	87.5% (21)	
	Participating in educational huddles has helped me become more efficient with rounding teaching service.	54.17% (13)	
	Participating in educational huddles has helped me improve my teaching during service.	70.83% (17)	
	Participating in educational huddles has helped me improve my skills in giving feedback.	62.5% (15)	
	Participating in educational huddles has helped me provide individualized coaching for learners.	70.83% (17)	
	Participating in educational huddles has helped improve the clinical care I provide to my patients.	54.17% (13)	
	Participating in educational huddles has helped increase my sense of belonging to the Division.	87.5% (21)	

Survey responses indicated a high degree of engagement and perceived relevance (Table 1). Faculty overwhelmingly viewed the huddles as a valuable use of time, with over 95% agreeing or strongly agreeing with that statement. Most respondents (79%) reported that issues raised during huddles reflected challenges they had encountered. Active participation was common, with 58% of hospitalists reporting they both sought and provided advice during an educational huddle.

Most respondents reported that participation in huddles improved key teaching‐related skills, including teaching on rounds (71%), providing feedback (63%), and individualized learner coaching (71%). More than half reported improved efficiency on rounds (54%) and improved clinical care (54%). Participation in the educational huddles was also associated with a strong sense of professional community, with 88% of respondents reporting they agreed or strongly agreed that participation increased their sense of belonging within DHM.

## DISCUSSION

Our results show that the weekly educational huddle was well‐received by academic hospitalists and supported their development as teachers. Specifically, hospitalists reported improvement in efficiency with rounding, teaching on service, giving feedback, and individualizing resident coaching. These reported gains aligned with the topics most frequently discussed during the huddles. We note that certain topics such as struggling learners were less frequently discussed, likely reflecting the expressed purpose of the huddle as a forum for peer coaching rather than reporting of learner deficits. Participation was also associated with an increased sense of belonging within the division.

Our findings suggest that the educational huddle facilitated the development of a CoP. A successful CoP requires three key components: joint enterprise, mutual engagement, and shared practice.^7^ The shared responsibility of supervising residents on ward rotations established a clear joint enterprise, which was reflected in faculty reports that the huddles clarified expectations for hospitalists. The in‐person format and emphasis on collaborative problem‐solving created robust mutual engagement. Most participants reported seeking or giving advice in the huddle, and more than three‐fourths of the group reported that discussions pertained to issues that they had encountered. We also note that the flexibility of the huddle structure allowed discussions to address immediate and evolving challenges, supporting just‐in‐time skill development. A shared practice that supports improving learning for resident physicians is the long‐term aim of the educational huddle. Although we did not specifically measure changes in observable teaching behaviors or learner outcomes, hospitalists self‐reported improvements across multiple domains. This self‐perceived improvement could underpin future change in behavior as suggested by self‐efficacy theory and a possible movement toward a shared practice centered on high‐quality resident education, which could be the subject of future investigation.

A CoP model for medical education has been previously suggested.^4^ CoP has also been utilized for faculty development to improve teaching.^5^, ^7^, ^8^ Here, we demonstrate the feasibility, acceptance, and early effectiveness of using a brief educational huddle to cultivate a CoP of clinical education within hospital medicine. An educational huddle offers several advantages for developing a CoP in hospital medicine. Huddles are familiar structures in hospital settings, lowering barriers to participation. By designing our huddle to solicit group input, we allowed for variation in participation from peripheral participation (seeking advice) to full participation (providing advice, informing norms and best practices), thus ensuring hospitalists with varying experience stay engaged. The semistructured format allowed for discussion to adapt quickly to respond to immediate needs. This works particularly well for hospital medicine, where the context of the learning environment in terms of workload, acuity, and team capabilities can vary widely; therefore, our educational huddle was uniquely designed to foster a CoP within hospital medicine.

We recognize several limitations to this innovation. The low response rate could result in a nonresponse bias. Our design of a weekly in‐person faculty educational huddle was possible as multiple faculty are located at the same site, but some programs may not be in the same situation or may have different rounding structures that could limit replication of our model. We had reliable access to dedicated meeting space, which may be a limitation for others. While we have data on self‐reported increase in teaching and coachingskills, application in practice and learner outcomes were not studied.

## CONCLUSION

An educational huddle can be a feasible, acceptable, and effective format for just‐in‐time development of teaching skills for hospitalists’ supervision of learners on ward rotations, with the added advantage of forming a CoP for iterative faculty development. Future work in this area that evaluates long‐term sustainability and measures effects on learner outcomes would be valuable.

## CONFLICT OF INTEREST STATEMENT

The authors declare no conflicts of interest.

## Supporting information

education huddle appendices.

## Data Availability

The data that support the findings of this study are available from the corresponding author upon reasonable request.
