# Supplementary material for: The implementation of an innovative just‐in‐time educational huddle for faculty development of academic hospitalists
Source: J Hosp Med. 2026 Sep 3;21(11):1318–21. doi: 10.1002/jhm.70436 (PMC13728521; doi:10.1002/jhm.70436)
Supplement: Supplementary file 1 [file JHM-21-1318-s001.docx]

Appendix 1:

Script followed by each teaching attending at the beginning of the huddle.

1. Overall assessment - how is the week going? How are the residents and students doing?
2. Brief update on rounding structure and duration for your team this week.
3. Are residents and students getting to conferences?
4. Have you set expectations and goals with your learners?
5. Is there anything you want input on in order to better coach your residents or students?
6. Are there major patient-related concerns that need to be addressed?

Appendix 2:

Exit instrument.

1. Date of huddle
2. How long did the huddle take (in minutes)?
3. How many team attendings were present?
4. What topics were discussed during the educational huddle?

Appendix 3:

Survey sent to teaching faculty to seek feedback on the huddles.

1. In the past 12 months approximately how many weeks of service have you done on a teaching team? [free text response]
2. What is the most rewarding aspect of working on resident teams for you? [free text response]
3. What is the most challenging aspect of working on resident teams for you? [free text response]
4. How often have you been able to attend the educational huddles when on resident teams? [Always - More than half - Less than half - Never]
4. When you attended, how often did you seek advice from the group? [Always - More than half - Less than half - Never]
4. When you attended, how often did you give advice on a topic raised in the group? [Always - More than half - Less than half - Never]
4. When you attended, how often did someone seek advice on an issue that you have also encountered? [Always - More than half - Less than half - Never]

Please rate your agreement with the following statements:

8. The educational huddles are a valuable use of my time during teaching service. [1 (strongly disagree) - 5 (strongly agree)]
8. Participating in educational huddles has helped me understand the expectations of attendings on teaching service. [1 (strongly disagree) - 5 (strongly agree)]
8. Participating in educational huddles has helped me become more efficient with rounding on teaching service. [1 (strongly disagree) - 5 (strongly agree)]
8. Participating in educational huddles has helped me improve my teaching during service. [1 (strongly disagree) - 5 (strongly agree)]
8. Participating in educational huddles has helped me improve my skills in giving feedback. [1 (strongly disagree) - 5 (strongly agree)]
8. Participating in educational huddles has helped me provide individualized coaching for learners. [1 (strongly disagree) - 5 (strongly agree)]
8. Participating in educational huddles has helped me improve the clinical care I provide to my patients. [1 (strongly disagree) - 5 (strongly agree)]
8. Participating in educational huddles has helped increase my sense of belonging to the Division. [1 (strongly disagree) - 5 (strongly agree)]

16. Number of years since residency training: [free text response]
16. Number of years as faculty teaching internal medicine residents (at any institution): [free text response]
16. Since starting medical school, have you had any formal training in health professions education (select all that apply): [Workshops, Peer Observation and Feedback, Faculty Development Course, Master’s Degree, Doctoral Degree, None of the Above, Other]
